# 

**Published:** 1916-07-01

**Authors:** 


### CONTENTS.

PAGE

**The Mobilisation of the Medical Profession** ... 317

**On Euthanasia** ... ... ... ... ... ... ... 318

**Hospital and Institutional News** ... ... ... 319

Charity Abuses *versus* Dead Committees—The Registration of War Charities—Our National Disasters—The New Volume of "Burdett's Annual"—Books of Reference in the Making—The Saturday Fund and Military Service—The Red Cross Emblem on Private Hospitals—Workshop Collections after the War—The War and the Saturday Funds—Physical Clinic for Disabled Soldiers—An Extraordinary Announcement—Concerts at the Front—Hospital Life in Local Newspapers—Doncaster's New Infirmary Scheme—An Objectionable Proceeding at Westcliff—The Milk Supply at Haslar—Some Large Contingent and Other Bequests—Miners' Phthisis in South Africa—This Week's Drug Market.

**The Nature of the Soul** ... ... ... ... 323

Some Primitive Ideas.

**A Medical Causerie** ... ... ... ... ... ... 324

**The Institutional Treatment of Consumption** 325

V.—The Disposal of Sputum.

**The Pioneer of the Sanatorium Idea** ... ... 326

**Florence Nightingale and the Royal National Pension Fund for Nurses** ... ... ... 329

Letters and Correspondence of Historic Value.

PAGE

**The Institutional Library** ... ... ... ... ... 327

Pye's Surgical Handicrafts—Diseases of the Nose and Throat, comprising Affections of the Trachea and Œsophagus—Occupations—Pharmacy and Dispensing—Children's Nursing—The Medical Annual, 1916—Letters from Another Battlefield—Macmillan and Another *v.* Nursing Press, Limited, and Others.

**Passing Events and Topics** ... ... ... ... 330

**The Matrons' and Sisters' Department** ... 331

The Royal British Nurses' Association.—II.
Round the Hospitals.

More Time for the College—"Mostly Thistles and Thorns"—Thoughtless Claimants in a Hurry—Self-Denial and Straight Conduct—Courtesy and the Royal Free Hospital—Matrons, Sisters, and Institutions.

**The Editor's Letter-Box** ... ... ... ... 333

**The Royal National Pension Fund for Nurses** 334

Some Remarkable Figures and Facts.

**Parliament and the Profession** ... ... ... 334

**From Across the Seas** ... ... ... ... ... 335

**Hospital Results in 1915** ... ... ... ... 335

**Welsh Education** ... ... ... ... ... ... 336

**Matrons' and Sisters' Appointments** ... ... 336

**Gifts to Hospitals** ... ... ... ... ... ... 336

**The Institutional Worker** ... ... (Suppl.) 1

